# Cardiovascular disease and impoverishment averted due to a salt reduction policy in South Africa: an extended cost-effectiveness analysis

**DOI:** 10.1093/heapol/czv023

**Published:** 2015-04-03

**Authors:** David A Watkins, Zachary D Olson, Stéphane Verguet, Rachel A Nugent, Dean T Jamison

**Affiliations:** ^1^Division of General Internal Medicine, Department of Medicine, University of Washington, Seattle, WA, USA,; ^2^Department of Medicine, Groote Schuur Hospital and the University of Cape Town, Cape Town, South Africa,; ^3^School of Public Health, The University of California, Berkeley, CA, USA,; ^4^Department of Global Health and Population, Harvard T.H. Chan School of Public Health, Harvard University, Boston, MA, USA,; ^5^Department of Global Health, University of Washington, Seattle, WA, USA and; ^6^Global Health Sciences, The University of California, San Francisco, CA, USA

**Keywords:** cardiovascular disease, economic evaluation, equity, financial risk protection, salt reduction, health care financing, South Africa, extended cost-effectiveness analysis

## Abstract

The South African Government recently set targets to reduce cardiovascular disease (CVD) by lowering salt consumption. We conducted an extended cost-effectiveness analysis (ECEA) to model the potential health and economic impacts of this salt policy. We used surveys and epidemiologic studies to estimate reductions in CVD resulting from lower salt intake. We calculated the average out-of-pocket (OOP) cost of CVD care, using facility fee schedules and drug prices. We estimated the reduction in OOP expenditures and government subsidies due to the policy. We estimated public and private sector costs of policy implementation. We estimated financial risk protection (FRP) from the policy as (1) cases of catastrophic health expenditure (CHE) averted or (2) cases of poverty averted. We also performed a sensitivity analysis. We found that the salt policy could reduce CVD deaths by 11%, with similar health gains across income quintiles. The policy could save households US$ 4.06 million (2012) in OOP expenditures (US$ 0.29 per capita) and save the government US$ 51.25 million in healthcare subsidies (US$ 2.52 per capita) each year. The cost to the government would be only US$ 0.01 per capita; hence, the policy would be cost saving. If the private sector food reformulation costs were passed on to consumers, food expenditures would increase by <0.2% across all income quintiles. Preventing CVD could avert 2400 cases of CHE or 2000 cases of poverty yearly. Our results were sensitive to baseline CVD mortality rates and the cost of treatment. We conclude that, in addition to health gains, population salt reduction can have positive economic impacts—substantially reducing OOP expenditures and providing FRP, particularly for the middle class. The policy could also provide large government savings on health care.

**Key Messages**

- The burden of cardiovascular disease (CVD) in South Africa is rising, and to address this, the government recently developed policies to reduce salt consumption in the population.
- We evaluated South Africa’s salt policy using extended cost-effectiveness analysis (ECEA), which models the health gains, financial risk protection and distributional effects of public policies.
- The salt reduction policy could reduce the burden of CVD by 11% yearly and avert catastrophic or impoverishing health expenditure in thousands of households, predominantly in middle-income groups.
- The policy could also save the government US$ 51.25 million yearly in subsidies for lower-income households, and thus, create fiscal space to invest further in CVD prevention.

## Introduction

The report of ‘The Lancet’ Commission on Investing in Health highlighted the key role that public policies can play in combating the rise of non-communicable diseases (NCDs) globally (Jamison *et al.* 2013). Population-based salt reduction strategies have been found to be a cost-effective approach to lowering the prevalence of hypertension and preventing cardiovascular disease (CVD) (World Health Organization [WHO] 2011). In South Africa, exposure to CVD risk appears to be rising, in part because of increases in hypertension prevalence and an aging population (Mayosi *et al.* 2012). Recent surveys have also highlighted the prevalence of high salt consumption (Charlton *et al.* 2005). In 2011, the South African Government proposed several CVD prevention targets, including lowering population salt intake to 5 g per person daily. The Government hopes to achieve this target by regulating salt in processed foods and coordinating media campaigns to lower discretionary salt use (Hofman and Tollman 2013).

The Government of South Africa also has economic reasons for attempting to reduce CVD risk. CVD is associated with premature mortality and long-term disability, often leading to absenteeism and reduced productivity that result in lower macroeconomic output (Marquez and Farrington 2013). CVD treatment in South Africa is also costly, and the government subsidizes health care for a large proportion of the population (McIntyre 2009). Despite subsidies, out-of-pocket (OOP) expenditures are significant and have a disproportionate impact in lower income groups (Harris *et al.* 2011) and rural, underserved areas (Goudge *et al.* 2009). High OOP expenditures are not unique to South Africa, however: studies of CVD costs in other developing countries demonstrate population groups with similar inability to pay (Kankeu *et al.* 2013). In these settings, catastrophic health expenditures (CHEs) are common, particularly for conditions such as CVD that are costly and less likely to be publicly financed (Huffman *et al.* 2011).

Recently, an analytic approach called ‘extended cost-effectiveness analysis’ (ECEA) was developed to assess the broader health system impacts of public policies (Verguet *et al.* 2015; 2015a,b). Whereas traditional cost-effectiveness analysis (CEA) estimates health impact per dollar spent, ECEA goes beyond CEA by estimating the socio-economic distribution of health gains and the financial risk protection (FRP) afforded by policies (Verguet *et al.* 2013). ECEA is thus intended to inform priority setting, particularly for diseases that have a socioeconomic gradient – for example, between wealthy and poor, women and men, urban and rural – or which are concentrated in marginalised ethnic groups or associated with high OOP expenditures (Jamison *et al.* 2013).

In South Africa, a preliminary analysis suggested that substantial health gains could be realized from modest salt reduction in targeted food groups (Bertram *et al.* 2012). The Government’s recently passed salt regulations reflect a new NCD target developed by the World Health Organization and provide a unique opportunity to apply the ECEA methodology to a health policy of growing relevance in low- and middle-income countries. The objective of the present study is to estimate the health and broader economic impact of South Africa’s salt policy targets.

## Methods

### Overview of model

The ECEA salt reduction model incorporates the following steps: (1) defining the population at risk of CVD due to high salt intake using current levels of salt consumption and blood pressure, then estimating (2) the impact of the salt reduction policy on population blood pressure levels, (3) the subsequent change in incidence and mortality from CVD, (4) the reduction in expenditures on CVD attributable to lower incidence, (5) the FRP provided by the policy and (6) the distributional impact of the policy by income quintile. The principal data inputs and their sources are listed in Table 1 and briefly described later. Drawing from methods developed for previous ECEAs (Verguet *et al.* 2015), we include equations in Supplementary S1 that describe how we modelled the health and economic effects of the salt policy.

**Table 1.** Main health-related inputs for the ECEA, including ranges used in sensitivity analysis

Data input	Mean value per income quintile					Range used in sensitivity analysis
	Q1	Q2	Q3	Q4	Q5	
Prior mean salt intake, g/day (Charlton *et al.* 2005)	7.8	7.9	7.9	8.0	8.6	7.3 to 10.5
Prior mean SBP, mmHg (SALDRU 2012)	131.2	133.7	134.1	137.2	132.6	N/A
Mean SBP change, mmHg						
Hypertensive individuals (He and MacGregor 2004)	−3.4	−3.4	−3.5	−3.6	−4.4	(−2.6) to (−6.6)
Mean SBP change, mmHg						
Normotensive individuals (He and MacGregor 2004)	−1.7	−1.7	−1.7	−1.8	−2.1	(−0.9) to (−3.9)
CVD death rate* per 100 000 population (IHME 2013)						
Stroke	1646	2079	1850	1823	1437	959 to 2849
IHD	1106	1424	1319	1310	1085	723 to 2073
HHF	704	881	778	760	591	305 to 1460
ESRD	88	110	104	101	90	51 to 186
Average CVD risk reduction achievable by policy (Lewington *et al.* 2002, Norman *et al.* 2007)						
Stroke	0.08	0.08	0.08	0.09	0.10	0.02 to 0.19
IHD	0.07	0.07	0.07	0.07	0.08	0.03 to 0.16
HHF	0.16	0.16	0.16	0.18	0.19	0.09 to 0.33
ESRD	0.16	0.16	0.16	0.18	0.18	0.09 to 0.33
CVD CFR						
Stroke (Feigin *et al.* 2009)	0.266	0.266	0.266	0.266	0.266	0.180 to 0.350
IHD, male (Mathers *et al.* 2004)	0.620	0.620	0.620	0.620	0.620	0.410 to 0.830
IHD, female (Mathers *et al.* 2004)	0.720	0.720	0.720	0.720	0.720	0.470 to 0.970
HHF (Damasceno *et al.* 2012)	0.155	0.155	0.155	0.155	0.155	0.124 to 0.194
ESRD (Ogeng'o *et al.* 2011)	0.230	0.230	0.230	0.230	0.230	0.184 to 0.288

SBP, systolic blood pressure; CVD, cardiovascular disease; IHD, ischemic heart disease; HHF, hypertensive heart failure; ESRD, end-stage renal disease. Q1, poorest quintile; Q5, wealthiest quintile.

*Death rate ranges reflect the highest and lowest death rate used for any of the five quintiles.

### Baseline characteristics of model population

The baseline characteristics of our model population were specified using Wave 3 of the National Income Dynamics Study (NiDS), a nationally representative panel survey of South Africans that was carried out in 2012 (SALDRU 2012). We used participants’ measured average systolic blood pressure and their reported age, sex and household income and divided them into five income quintiles (Table 1 and Supplementary S2). We then used data from a nutrition survey in Cape Town to assign each cohort member an average daily salt intake based on his or her ethnicity (Charlton *et al.* 2005). For the subsequent model steps, we calculated all health and economic effects as weighted mean effects in a cohort of 1 million adults 40 years or older, divided into five income quintiles.

### CVD averted by salt reduction

We calculated the mean change in salt consumption from current levels to the government’s target of 5 g per day across each quintile. We then estimated the impact of sustained salt reduction on long-term blood pressure using regression coefficients from a recent meta-analysis (He and MacGregor 2004).

We defined four major hypertension-related CVD outcomes of relevance: stroke, ischemic heart disease (IHD), hypertensive heart failure (HHF) and end-stage renal disease due to hypertension (ESRD). We matched age- and sex-specific death rates from each of these outcomes to NiDS respondents and estimated the weighted average CVD risk in each quintile (IHME 2013). We then calculated the incidence of each outcome using regional case-fatality rates (CFRs) (Mathers *et al.* 2004; Feigin *et al.* 2009; Ogeng'o *et al.* 2011; Damasceno *et al.* 2012). We then estimated the policy’s effect on reducing the incidence and mortality from the four CVD outcomes using age- and sex-specific hazard ratios (Lewington *et al.* 2002; Norman *et al.* 2007). Because we used 1-year mortality and incidence rates, we present all health and economic effects as yearly estimates.

### Calculation of OOP costs

**Figure 1.**
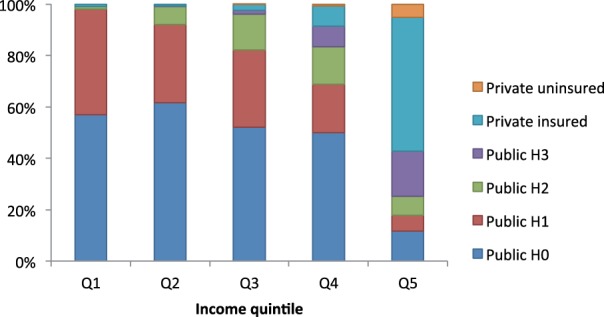
Payment for health services in South Africa: mix of payer categories in a cohort of 1 million adults aged 40 years or older, based on data from the NiDS, Wave 3. H0, H1, H2 and H3 refer to sliding scale payer categories at public facilities where H0 is fully subsidized, H1 and H2 are partially subsidized and H3 is unsubsidized. Q1, lowest income quintile; Q5, highest income quintile. See Supplementary S2 for further details of how payer mix was estimated

South Africa’s health system is a hybrid of public and private facilities (McIntyre 2009), and OOP fees are determined on a sliding scale in the former (Western Cape Government 2013) and by the presence or absence of health insurance in the latter (Mediclinic 2013). Hence, the OOP cost of CVD care in South Africa varies widely based on facility type, income level and eligibility for subsidies. Generally, lower income groups pay less for care in the public sector because they are eligible for larger subsidies, and older adults and the poor pay almost nothing OOP. On the other hand, higher income groups are not eligible for these subsidies in the public sector, and a higher proportion of them seek care in private facilities, which are much more expensive. The payer mix used in our model is illustrated in Figure 1, and the algorithm used to assign cohort members to payer categories is described in Supplementary S3.

We compiled a list of cost ingredients for treating hypertension and each CVD outcome based on local guidelines (Bryer *et al.* 2010; Seedat *et al.* 2012), registry data (Damasceno *et al.* 2012; Schamroth 2012) and consultation with local specialists. For each ingredient, we used published fees for public (Western Cape Government 2013) and private (Mediclinic 2011) facilities and converted these to OOP costs based on payer category subsidy rates. CVD outcome and hypertension costs by quintile are listed in Table 2. The variation in hypertension and CVD costs reflect the impact of decreasing levels of subsidy with increasing income as well as the increasing use of private care by wealthier households. The variation in costs also reflects disease-specific differences in management; e.g. IHD and ESRD include hospitalization and expensive procedures, whereas hypertension is primarily managed as an outpatient with inexpensive medications. The details of the costing methodology are given in Supplementary S4.

**Table 2.** Main OOP cost inputs for the ECEA, including ranges used in sensitivity analysis

Yearly cost*	Mean value per income quintile				
	Q1	Q2	Q3	Q4	Q5
Hypertension	$2	$4	$7	$12	$24
(Range)	(1–5)	(2–7)	(3–13)	(6–23)	(12–48)
Stroke	$18	$39	$100	$288	$863
(Range)	(10–42)	(21–82)	(54–215)	(162–649)	(565–2260)
IHD	$22	$52	$128	$338	$992
(Range)	(13–53)	(28–111)	(71–283)	(201–805)	(730–2919)
HHF	$15	$29	$74	$212	$638
(Range)	(8–31)	(15–59)	(37–149)	(108–432)	(332–1333)
ESRD	$151	$221	$388	$630	$1731
(Range)	(75–301)	(111–442)	(194–262)	(315–1261)	(866–3462)

OOP, out-of-pocket; IHD, ischemic heart disease; HHF, hypertensive heart failure; ESRD, end-stage renal disease. Q1, poorest quintile; Q5, wealthiest quintile. Currency is 2012 USD. For details of the costing methodology, assumptions and data sources, please see Supplementary S3.

*All costs are reported in 2012 USD.

### OOP expenditures and government subsidies averted

We estimated the total OOP expenditures averted during the first year after incident CVD in our cohort using the expected cost and reduction in incidence of the four CVD outcomes. We estimated the government subsidies averted by calculating the total amount of subsidy for each case of CVD averted in each payer category. We also estimated the reduction in government subsidies for hypertension treatment.

### Estimation of the cost of the salt reduction policy

As mentioned previously, South Africa’s salt reduction policy contains two components: (1) an informational campaign to reduce discretionary salt use and (2) mandatory regulation of maximum salt content in commercially produced foods in six key groups. Planning for the former component is currently in preparation and has been estimated to cost the Government US$ 500 000 over 6 months (K Steyn, personal communication—August 2014). To estimate the annual cost of the policy, we assumed a 10-year policy cycle in which the cost of this campaign was distributed evenly as US$ 50 000 per year. To this cost, we added the cost of enforcing the salt regulations as described later. The reasoning for annualizing this up-front cost was to align the average costs of the programme with the timing of the other health and economic outcomes.

Although regulations on salt content in processed foods were approved in 2013, they will not be enforced until 2016, giving the food industry time for product reformulation. Hence, the infrastructure for monitoring and enforcing these regulations has not been developed nor have local costs been estimated. Given these limitations, the current analysis used data from a recent study in the Eastern Mediterranean region (Mason *et al.* 2014) to estimate the South African Government’s cost of oversight and enforcement. Using population estimates (UN 2014) and total public sector costs reported for Turkey, Syria and Tunisia (Mason *et al.* 2014), we calculated an average per capita regulatory cost, which we multiplied by South Africa’s population size. This cost was estimated at US$ 5.72 million over 10 years. To this regulatory cost we added US$ 25 000 per year, the estimated cost of a small survey to measure salt consumption and monitor the policy’s effect (K Steyn, personal communication—August 2014). The total cost of the policy to the South African Government was estimated at US$ 677 337 per year or ∼US$ 0.01 per capita per year.

Finally, it is possible that the food industry’s costs of reformulating their products could be passed along to consumers, and that these costs could adversely affect lower-income groups. To assess the potential impact of shifting reformulation costs to consumers, we used data from the previously mentioned Eastern Mediterranean study (Mason *et al.* 2014) to estimate the private sector cost of reformulating food products. Again, we used population estimates (UN 2014) and total private sector costs reported for Turkey, Syria and Tunisia (Mason *et al.* 2014) to calculate an average per capita cost of reformulation, which we multiplied by South Africa’s population size. This cost was estimated at US$ 137 million over 10 years. Assuming 100% of the cost would be passed onto consumers; the yearly cost of US$ 13.7 million would translate into an increase in food expenditures of US$ 0.25 per capita.

### Financial risk protection

We estimated FRP using two different metrics: cases of CHE averted and cases of poverty averted. To remain consistent with other South African studies (Goudge *et al.* 2009; Harris *et al.* 2011), we defined CHE as any case of CVD expenditure exceeding 10% of total yearly household income. We defined CVD expenditure as impoverishing if it reduced individual income below the poverty line. We used a published poverty line (Statistics South Africa 2008) of US$ 78 (2012) per person monthly, which implies that 37·5% of respondents (most of quintiles one and two) currently live in poverty.

### Sensitivity analysis

We performed a univariate sensitivity analysis on key parameters to test their impact on our results. The ranges used in the analysis are listed in Tables 1 and 2 and their derivation described in Supplementary S5. We also tested the sensitivity of poverty cases averted to two lower poverty lines, US$ 36 and US$ 53 per person monthly (Statistics South Africa 2008). To place our FRP estimations in context, we reassigned all subsidized cohort members to the full OOP cost of care and re-calculated the CHE and poverty cases averted; the difference between these results and the original CHE and poverty cases reflects the current FRP provided by the government. Finally, while our model assumed all patients receive care, we tested the sensitivity of expenditures averted and FRP provided to proportional decreases in service utilization.

### Other methods

We analysed the survey data and ran the model in STATA version 13·0 (College Station, TX). All costs were inflated to and reported in 2012 US dollars (USD). Because this study only estimated 1-year expenditures on incident CVD, costs were not discounted. We used publicly accessible data, so no additional ethics approval was required according to the criteria of the University of Washington Institutional Review Board.

## Results

### Health gains

The major health and economic gains that could be achieved by the policy are summarized by income quintile in Table 3. In a model cohort of 1 million South African adults, the policy averted 403 deaths and 1680 cases of CVD per year. The distribution of deaths averted was 39% stroke, 34% HHF, 23% IHD and 4% ESRD. The distribution of cases averted was 35% stroke, 52% HHF, 8% IHD and 5% ESRD.

**Table 3.** The ECEA dashboard: major health and economic impacts of salt reduction in a cohort of 1 million South Africa adults

	Q1	Q2	Q3	Q4	Q5	Total
Programme costs per capita						
To government	$0.01	$0.01	$0.01	$0.01	$0.01	$0.01
To households*	$0.25	$0.25	$0.25	$0.25	$0.25	$0.25
Programme savings per capita						
To government	$2.62	$3.27	$2.95	$2.92	$0.85	$2.52
To households	$0.02	$0.05	$0.07	$0.23	$1.11	$0.29
Health and FRP gains						
CVD deaths averted	69	86	79	86	83	403
CHE cases averted	6	12	25	52	80	175
Poverty cases averted	0	81	35	27	2	145
Total government savings	$524 980	$654 500	$590 200	$583 400	$170 780	$2 523 850
Total OOP savings	$3750	$9920	$13 580	$45 740	$221 860	$294 860

CVD, cardiovascular disease; OOP, out-of-pocket; CHE, catastrophic health expenditure. Q1, poorest quintile; Q5, wealthiest quintile. All costs, savings, and health and FRP gains are per year. Currency is 2012 USD.

*Assumes that consumers bear 100% of the cost of food product reformulation (worst case scenario)

**Figure 2.**
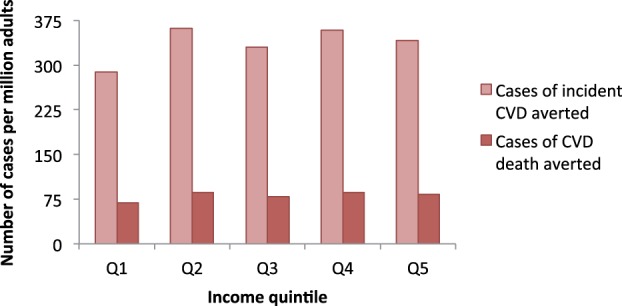
Distribution of potential deaths and incident cases of CVD averted by a salt reduction policy in a cohort of 1 million South African adults aged 40 years or older. Q1, lowest income quintile; Q5, highest income quintile

Analysed by income quintile, health gains were fairly evenly distributed, except for a slightly lower impact in quintile one (Figure 2). The age structure of this quintile was skewed towards younger adults than the other quintiles, and it was also comprised of more black African members, whose salt consumption was lower than other groups (Supplementary S2). Hence, the overall risk of CVD and impact of reducing salt intake in this group was lower.

### Private expenditures averted and policy costs

In total, approximately US$ 295 000 per year in OOP expenditures on CVD were averted in the cohort, ranging from US$ 0.02 per capita in the poorest quintile to US$ 1.11 per capita in the richest quintile (Table 3). Most of the total and per capita expenditures averted were in quintiles four and five, because wealthier individuals were much more likely to seek more-expensive private care or receive unsubsidized care in public facilities. Given the high rates of private insurance in quintile five, the OOP savings would most likely be realised as reduced insurance premiums for all individuals rather than as direct reductions in payments for medical care among the ill. On average, the potential OOP cost of reformulating food products (US$ 0.25 per capita) was slightly less than the expected reduction in OOP expenditures on CVD (US$ 0.29 per capita); however, in the lower income groups, this cost was greater than the expected reduction in OOP expenditures on CVD (Table 3).

### Government subsidies averted and policy costs

The financial benefit of salt reduction to the South African Government was much larger than the benefit to households. Approximately US$ 2.52 million in government subsidies on CVD care per year (for stroke, IHD, HHF and ESRD combined) were averted in the cohort, ranging US$ 0.85 per capita in quintile one to US$ 3.27 per capita in quintile five. In contrast to the private expenditures, most government savings were on individuals in quintiles one through four. The vast majority of savings were on care for stroke and HHF, which, though less expensive on average than IHD and ESRD, were more common. We calculated the reduction in government subsidies on hypertension treatment to be an additional US$ 1.197 million per year (US$ 1.20 per capita). The per capita cost of the policy to the Government was ∼US$ 0.01 and was vastly outweighed by the savings on CVD care expenditures, which ranged from US$ 0.85 per capita in the quintile five to US$ 3.27 per capita in quintile two or US$ 2.52 per capita overall (Table 3).

### FRP provided

**Figure 3.**
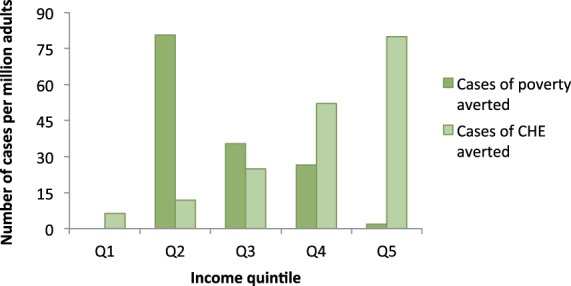
Distribution of potential FRP from CVD expenditure provided by a salt reduction policy in a cohort of 1 million South African adults aged 40 years or older. FRP is measured separately as cases of CHE or impoverishing health expenditure. Q1, lowest income quintile; Q5, highest income quintile

When we estimated FRP using the CHE metric, 175 cases of CHE per year were averted in the cohort; using the poverty line metric, 144 cases of poverty per year were averted. As in the expenditure analysis, the higher proportion of HHF and stroke cases was the main driver of the FRP estimates. Figure 3 illustrates the distributional impact of the FRP provided by the policy. No cases of poverty were averted in the poorest quintile, since everyone in this quintile was already impoverished; however, large benefits were realized in the middle-income quintiles, particularly quintile two, where individuals live relatively nearer to the poverty line. More CHE cases were averted in the upper income quintiles; again, these results were largely driven by more expensive care accessed by the wealthy.

To put these results in context, we also calculated the current FRP provided by the government for CVD care attributable to salt intake. Without the large government subsidies on care, 1243 cases of CHE and 798 cases of poverty would be averted by the policy. Thus, the policy in itself averts an additional 12% of cases of CHE and 15% cases of poverty beyond the government’s existing subsidy efforts.

### Sensitivity analysis

**Figure 4:**
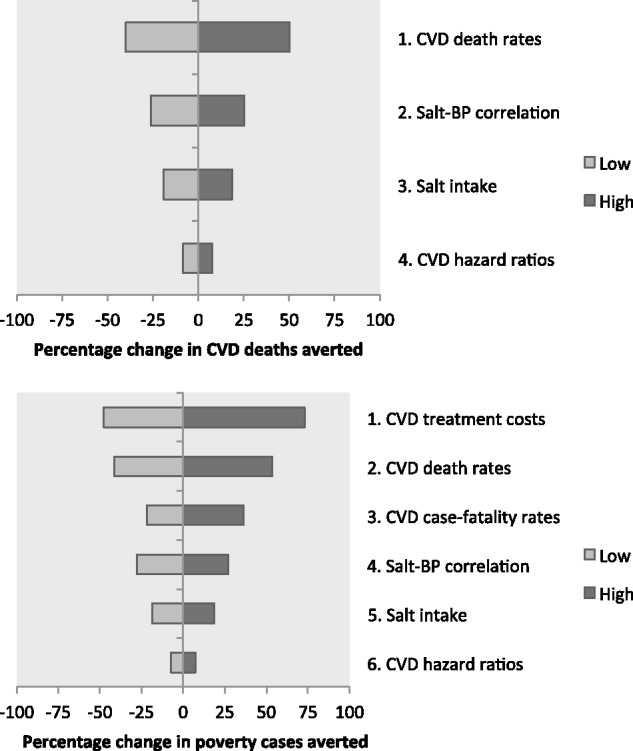
Example sensitivity analyses of model results. The top panel demonstrates the percent increase (darker shading) or decrease (lighter shading) in deaths averted when various model inputs are increased or decreased, respectively. Similarly, the bottom panel demonstrates the percent increase or decrease in the cases of poverty averted when various model inputs are changed. The bottom panel contains additional inputs—treatment costs (1) and CFRs (3)—because these are required for the calculation of poverty cases averted but not for the calculation of deaths averted. Sensitivity analyses of other results followed similar trends and are provided in Supplementary S5. CVD, cardiovascular disease; BP, blood pressure

Two examples of univariate sensitivity analyses of key outcomes are provided in Figure 4. In general, the health gains were more sensitive to variation in CVD death rates, the correlation between salt intake and blood pressure, and overall salt intake levels, and they were less sensitive to variation in the hazard ratios that we used to calculate the reduction in CVD mortality. As might be expected, the economic gains were most sensitive to variation in cost of CVD care; their sensitivity to variation in the epidemiologic inputs was somewhat lower and followed patterns similar to the analysis of health gains. Tornado diagrams for other key outcomes are provided in Supplementary S5.

In addition to the main sensitivity analysis, we also modelled FRP using lower poverty lines of US$ 53 and US$ 36 per person monthly. Under these assumptions, the policy reduced the poverty cases averted by 56 and 53%, respectively (baseline poverty rates of 22.7 and 10.5%, respectively). Because of the linear relationships between salt consumption, CVD risk and FRP, a lesser impact on salt consumption (e.g. 1.5 g instead of ∼3 g) would lead to approximately proportional reductions in the health and economic gains (e.g. 50% fewer deaths or cases of poverty averted), all else being equal. Similarly, when we modelled lower rates of service utilization, cases of CHE or poverty averted and government subsidies averted were reduced by approximately the same percentage; e.g. if 15% of individuals did not seek care, FRP and government subsidies would be reduced by ∼15%. Of note, the health gains were unchanged in the scenario of lower utilization, since the policy’s effects covered the entire population.

## Discussion

We analysed the economic impact of a real-world salt reduction policy at a time when targets similar to South Africa’s are rapidly gaining traction globally (WHO 2011). We show that an NCD policy can improve health in all income groups, though with different economic effects in each group. Additionally, we demonstrate substantial cost savings to a government that already heavily subsidizes health care.

Consistent with prior models (Bertram *et al.* 2012; Institute of Medicine 2013), our study demonstrates that considerable reductions in CVD risk in South Africans could be achieved by reducing salt consumption. The most recent South African census estimates 13.77 million adults over the age of 40 (Statistics South Africa 2011). Our cohort estimates would thus translate into approximately 5600 deaths and 23 000 cases of CVD averted yearly in the current South African population. Viewed another way, the policy could avert up to 11% of the 50 000 deaths from the four major CVD outcomes in South Africa in 2010 (IHME 2013) and would be a key piece of the so-called ‘25 by 25’ agenda for reducing NCDs in South Africa (Mayosi *et al.* 2012).

Although many salt reduction models focus solely on IHD and stroke outcomes, we included HHF in our model because it is the most common hypertension-related CVD outcome in sub-Saharan Africa (Sliwa *et al.* 2008). Much of the overall health and economic impact of salt reduction in our model—between 30 and 50%, in fact—was mediated through the reduced incidence of HHF. Future analyses of CVD in the African region should regard this under-appreciated condition as a key NCD target.

In addition to the health benefits, we also assessed various economic effects of the policy. Based on census data, we estimate that US$ 4.06 million in OOP expenditures on CVD could be averted. Lower-income households would have less to gain: the poorest quintile would end up paying US$ 0.25 per capita yearly but only (on average) save US$ 0.02 in OOP expenditures on CVD. On the other hand, this would translate into a modest 0.13% increase in food expenditures. (By comparison, the wealthiest quintile would also pay US$ 0.25 per capita yearly and save US$ 0.29 in OOP expenditures on CVD. This would translate into a 0.02% increase in food expenditures). Furthermore, from a societal perspective this imbalance of costs could still be acceptable given the reduction in CHE and cases of to a large number of lower-income households. Future research should seek to assess whether such tradeoffs are acceptable to South Africans.

The financial benefit of salt reduction to the South African Government would be substantial given the large subsidies on CVD care that are currently provided for lower income groups. Based on census data, we estimated that US$ 34.75 million in government subsidies on CVD care and US$ 16.50 million in government subsidies on hypertension care could be averted yearly. The sum total of hypertension and CVD subsidies averted, then, could be US$ 51.25 million total (US$ 0.99 per capita) yearly. To put these numbers into context, the Government currently spends US$ 308.70 per capita on health (WHO 2013), so even after accounting for the cost of the policy, annual government expenditures on health could be reduced by 0.32%. Our model thus demonstrates that investing in CVD prevention can create the fiscal space to invest in other NCD policies.

The novel aspect of ECEA, and the most important contribution of this study to the existing literature on salt reduction, is the estimation of FRP and distributional consequences of the salt policy. Although the health gains were fairly uniform throughout the population, the middle three income quintiles—which include the vast majority of cases—experienced an increase in FRP on CVD expenditure by reducing their salt consumption. In fact, we estimate that in the entire South African population, 2410 cases of CHE and 1988 cases of poverty could be averted yearly by reducing salt consumption. However, because of the progressive nature of the South African health system, the additional FRP ‘purchased’ by the government by investing in salt reduction is likely to be modest compared with countries with larger OOP contributions to total healthcare expenditure. Thus, application of the ECEA approach to NCD policies in countries with different health financing systems will likely yield very different results, making it a useful tool at the local level for decision-makers interested in health equity.

It is not clear whether the South African Government’s ambitious policy target can be reached using only the proposed combination of education and regulation. Assuming an average current salt consumption of ∼8 g per person daily, the policy seeks to reduce consumption by ∼38%. Cost-effectiveness studies in other countries have examined salt reduction packages that assume a 15–30% reduction from baseline (Asaria *et al.* 2007; Mason *et al.* 2014). On the other hand, the South African targets were set using the best available data on food consumption patterns in different ethnic groups and were explicitly tailored towards the most commonly consumed food groups (Hofman and Tollman 2013), and fact that the regulations are mandatory instead of voluntary (He *et al.* 2014) will likely increase the impact of the policy. Furthermore, the experience of countries like Finland, Japan and UK does suggest that sustained reductions in population salt intake on the order of 4 to 5 g per person daily are possible, though they may take years to reach (WHO 2011; He *et al.* 2014). In a sensitivity analysis, we found that a 50% lower effectiveness (∼1.5 g less salt per person daily) would lead to 50% of the health impact, but the cost savings would remain substantial, particularly from the government perspective.

Our analysis has several important data limitations. First, the epidemiologic data have a degree of uncertainty, which we attempted to place bounds on by performing a sensitivity analysis. Nevertheless, nationally representative salt consumption data and local cohort studies of CVD would strengthen our results. Second, because of data limitations, we could not model potential net harms to reduced salt consumption in select subpopulations, e.g. individuals with advanced heart failure (Institute of Medicine 2013); these harms, while relatively small, could reduce the net health gains. Third, the actual OOP cost of CVD care is not known, and true costs could be much higher or lower, depending on co-morbidities and health service utilization patterns. In light of the rapid increase in NCDs in South Africa, there is urgent need to gather empirical cost estimates on CVD to inform economic models and allocate resources. Finally, since there were no local data on the cost of the policy, we had to extrapolate costs from other countries that have formally estimated the cost of similar regulations. It is possible that the true cost of regulation in South Africa could be higher or lower than in these other countries.

Our results should also be interpreted with some caution. Our analysis assumes that the salt policy is effective across income groups. Careful monitoring and evaluation of the effectiveness of the regulations and campaigns should be undertaken; these efforts can inform policy decisions in other countries considering packages of NCD interventions. Additionally, from an economic perspective, we were not able to model FRP by other important metrics such as rates of distress financing (Kruk *et al.* 2009). Nor were we able to estimate potentially significant changes in productivity. Data permitting, future analyses could incorporate these sorts of economic effects.

## Conclusion

Shifting population salt consumption to a target distribution of 5 g per person daily could avert approximately 5500 deaths and 23 000 cases of CVD per year in the South African population. The Government could save up to US$ 51.25 million in subsidized care for poorer individuals, which suggests that on balance the policy would be cost-effective and perhaps cost saving. It is unclear what impact reformulating food products will have on food prices among the poor, however even in the worst case scenario, the increases would likely be modest compared with current household expenditures. Preventing CVD could also provide substantial FRP to individuals at risk of catastrophic and impoverishing OOP expenditure, particularly in the middle class. There is a need for more empirical data to quantify the OOP cost of CVD care in developing countries and to develop models that can estimate the long-term health and economic impact of preventing chronic, NCDs.

## Supplementary Material

Supplementary Data
