# Supplementary material for: Cardiovascular disease and impoverishment averted due to a salt reduction policy in South Africa: an extended cost-effectiveness analysis
Source: Health Policy Plan. 2015 Apr 3;31(1):75–82. doi: 10.1093/heapol/czv023 (PMC4724166; doi:10.1093/heapol/czv023)
Supplement: Supplementary Data [file supp_31_1_75__index.html]

Cardiovascular disease and impoverishment averted due to a salt reduction policy in South Africa: an extended cost-effectiveness analysis — Cardiovascular disease and impoverishment averted due to a salt reduction policy in South Africa: an extended cost-effectiveness analysis — Supplementary Data 

# Cardiovascular disease and impoverishment averted due to a salt reduction policy in South Africa: an extended cost-effectiveness analysis

## Supplementary Data

files

**Files in this Data Supplement:**

- Supplementary Data - doc file
