# Supplementary material for: Cardiovascular disease and impoverishment averted due to a salt reduction policy in South Africa: an extended cost-effectiveness analysis
Source: Health Policy Plan. 2015 Apr 3;31(1):75–82. doi: 10.1093/heapol/czv023 (PMC4724166; doi:10.1093/heapol/czv023)
Supplement: Supplementary Data [file supp_czv023_Salt_ECEA_supplement_revised_2.doc]

**Supplement S1. Extended cost-effectiveness analysis calculations.**

As described in the main text, we used individual-level survey data to estimate the health and economic effects of the salt policy by quintile. We report health and economic effects per quintile, so respondents were characterized by income quintiles
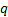
 based on household income per capita,
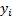
. Survey weights for each respondent were used in conjunction with STATA’s mean function to estimate weighted mean effects by quintile.

**Deaths and cases of CVD averted**

Risk of each of the CVD outcomes is age- and sex-specific, so we assigned death rates and hazard ratios to each survey respondent
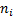
 based on these parameters. The number of deaths averted per quintile from a given CVD outcome (
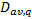
) is given as:


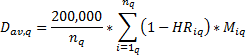


Where
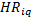
 is the age- and sex-specific hazard ratio for the *i^th^* individual in wealth quintile *q* of the cohort,
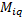
 is the age- and sex-specific mortality rate from stroke or IHD for that individual, and
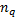
is the number of individuals in the NiDS who represent the 200,000-member quintile.

The number of CVD cases averted per quintile (
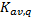
) by the salt reduction policy is given as:


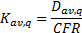


Where
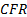
 is the case-fatality rate from any of the CVD outcomes.

**Private expenditures and government subsidies averted**

OOP costs for the four CVD outcomes are also payer- and disease-specific, so we assigned each respondent
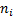
 we assigned an OOP cost based on his or her payer category. The reduction in OOP expenditures per quintile from a given CVD outcome (
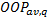
) is given as:


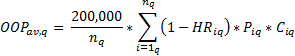


Where
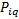
 is the age- and sex-specific incidence of stroke or IHD and is given as:


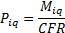


And
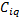
 is the OOP cost of the CVD outcome to the *i^th^* individual in income quintile *q* of the cohort.

The reduction in government expenditures per quintile (
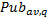
) due to the salt reduction policy is given as:


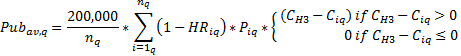


Where
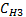
is the OOP cost of stroke of the CVD outcome to individuals in public payer category H3.

**Financial risk protection provided**

The number of cases of catastrophic health expenditure (i.e., exceeding 10% total yearly household income) averted per quintile from a given CVD outcome (
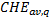
) is given as:


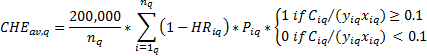


And
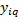
 is yearly household income per capita and
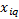
 is household size.

Similarly, the number of cases of poverty averted per quintile (
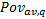
) by the salt reduction policy is given as:


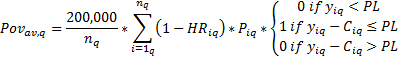


Where
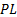
 is the poverty line.

**Supplement S2. Age structure and ethnic composition of the cohort by income quintile.**

**Table 1.** Age structure of the cohort by income quintile.

| **Age group** | **Q1** | **Q2** | | **Q3** | | **Q4** | | **Q5** | |
| --- | --- | --- | --- | --- | --- | --- | --- | --- | --- |
| 40-44 years | 18 | 16 | | 19 | | 16 | | 22 | |
| 45-49 years | 19 | 16 | | 15 | | 16 | | 20 | |
| 50-54 years | 19 | 14 | | 14 | | 16 | | 17 | |
| 55-59 years | 15 | 14 | | 15 | | 12 | | 14 | |
| 60-64 years | 10 | 12 | | 12 | | 14 | | 10 | |
| 65-69 years | 6 | 8 | | 9 | | 10 | | 6 | |
| 70-74 years | 5 | 9 | | 8 | | 7 | | 5 | |
| 75-70 years | 3 | 5 | | 4 | | 4 | | 3 | |
| 80+ years | 3 | 5 | | 6 | | 4 | | 3 | |
|  |  | |  | |  | |  | |  |

Values are given as percentages.

**Table 2.** Ethnic composition of the cohort by income quintile.

| **Ethnic group** | **Q1** | **Q2** | **Q3** | **Q4** | **Q5** |
| --- | --- | --- | --- | --- | --- |
| African | 93 | 89 | 79 | 75 | 54 |
| Mixed ancestry | 7 | 10 | 20 | 22 | 19 |
| Asian/Indian | 0 | 0 | 1 | 2 | 4 |
| White | 0 | 0 | 0 | 2 | 23 |
|  |  |  |  |  |  |

Values are given as percentages.

**Supplement S3. Determination of payer mix for each income quintile.**

As described in the main text, health care in South Africa is delivered through both public and private facilities. Out-of-pocket payments in public facilities are calculated based on a sliding scale of income and eligibility for free care as described below. Patients carrying insurance are charged co-pays according to the care received. Uninsured patients who wish to receive private care are only treated in private facilities if they have means to pay the full cost of care. Many such individuals are transferred to public facilities if they do not have means to pay.

In our model, “uninsured” cohort members are those who, according to the NiDS (SALDRU 2012), reported a preference for private facilities, do not carry health insurance, and have sufficient income to pay the full cost of private care for stroke or IHD. Similarly, “insured” individuals are those who reported a preference for private facilities but carry health insurance and are thus eligible to pay only co-payments.

Private care patients make up a small percentage of our cohort, with most individuals receiving care at public facilities. These facilities use a sliding scale classification system of H0, H1, H2, or H3 and charge patients at the appropriate payer rate for each item or service consumed. In our model, individuals were classified as H0-H3 if they reported preference for public facilities (according to the NiDS); or if they reported a preference for private facilities but had neither health insurance nor sufficient income to pay the full cost of private care for CVD.

Individuals eligible for H0 status (free care) include the elderly, those receiving child support, veterans, care-dependent persons, individuals with permanent disabilities, individuals in foster care, and the formally unemployed. These persons receive care free of charge in public hospitals if they can provide proof of their exempt status.

H1 individuals receive highly subsidized care upon proof of household income less than 50,000 rand per year (US$ 6095 in 2012; 1 rand = 0.1219 dollars). This is also the default payer category for individuals who cannot provide proof of income or H0 status at the point of care. H2 individuals receive partially subsidized care upon proof of household income between 50,000-100,000 rand (US$ 6095-12,190) per year. H3 individuals pay full hospital fees.

Figure 1 in the main text illustrates the proportion of individuals in our cohort who pay the rates described above. Most individuals in the lower income quintiles receive free care or pay highly subsidized rates. Conversely, there is a high demand for private care in the two highest income quintiles.

Please see the main text for references on the structure of the South African health care system. Please see Supplement S3 for details of the costing methodology for cardiovascular disease treatment in each payer category.

**Supplement S4. Costing methodology.**

To date, there are no empirical studies on the out-of-pocket cost of CVD care in South Africa. However, user fee schedules are publicly available both for public facilities and for some private facilities (Mediclinic 2013; Western Cape Government 2013). We modeled the average OOP cost of CVD care in each payer category for each of our four CVD outcomes: stroke, IHD, HHF, and ESRD.

We first developed a list of cost ingredients based on published treatment guidelines as referenced in the main text. Where ambiguity existed as to treatment standards, we consulted local specialist physicians to determine standard practices. We acknowledge that many patients might not receive best-practice care, particularly patients in less-resourced facilities, thus our ingredients lists are somewhat idealized. We also acknowledge that the ingredients approach may underestimate treatment costs because comorbidities and complications are not included. Finally, we assumed a uniform distribution of incident CVD over the entire year, such that the average individual would pay for six months of chronic outpatient treatment in addition to acute CVD events.

**Facility costs**

For acute stroke, heart attack, and heart failure treatment, we assumed that all patients would be taken to the nearest health facility by emergency medical services. All patients would be triaged in an emergency department. All stroke and IHD patients would be admitted to an intensive care unit (ICU) for 24 hours for stabilization and then would spend another five or four days on a general ward, respectively (K. Moeketsi, personal communication – Dec 2012). We estimated that 10% of HHF patients would be admitted to an ICU for cardiogenic shock and be stabilized over two days, then spend another five days on a general ward. The remaining 90% of HHF patients would spend seven days on a general ward.(Damasceno et al. 2012)

There are no studies on inpatient management of chronic kidney disease in South Africa. We assumed that ESRD patients would incur the majority from of day admissions to a renal dialysis unit (three times weekly) rather than from acute kidney injury hospitalization, thus we only considered outpatient facility and provider costs for ESRD. Of note, we did not include dialysis costs for ESRD cases over 60 years, as such individuals are not be eligible for long-term dialysis (B. Rayner, personal communication – Nov 2013).

For outpatient care, we assumed all patients would incur six months of secondary prevention costs following initial hospitalization. We thus included two primary health clinic assessments in the outpatient fees. Additionally, we recognized that some patients would receive care at level two and level three facilities, which are more expensive. We thus used survey data to create weighted average costs based on the proportion seeking care at each level (Harris et al. 2011).

**Provider costs**

For inpatient admissions, we assumed that all patients would be evaluated by an emergency department physician, an intensive care specialist (where applicable), and a general ward physician. When sedation was required for procedures, we included anesthesiologist consultation fees. We further assumed that IHD patients would receive a cardiology consultation and stroke patients would receive physical and occupational therapy services. We included technician fees and radiologist consulting fees for imaging studies. We also included nursing fees where appropriate.

For outpatient care, in addition to primary care assessments for all patients, we assumed that IHD patients would also be referred to a cardiologist once and that that ESRD patients would be assessed by a nephrologist at least once monthly.

**Diagnostic and treatment costs**

The fee schedules above outline pharmacy dispensary fees but not the cost of medications themselves. In order to calculate drug costs, we obtained price lists for all standard generic CVD medications from Groote Schuur Hospital (W. Bryant, personal communication – Dec 2012) and the University of Cape Town Private Academic Hospital (T. Ferger, personal communication – Feb 2013) for public and private facilities, respectively.

For stroke patients, we assumed 70% of strokes were ischemic and the remaining 30% hemorrhagic (Mudzi et al. 2012). We assumed that all patients would receive appropriate neuroimaging (CT scan and/or cerebral angiography) and that 1% of patients would be eligible for fibrinolytic therapy with alteplase (B. Mayosi, personal communication – Dec 2012). We included electrocardiography, carotid duplex ultrasonography, and transthoracic echocardiography in the routine stroke workup. We included the cost of aspirin and hydrochlorothiazide for both acute and chronic stroke treatment, and we included the cost of simvastatin and warfarin for select patients with lipid disorders or atrial fibrillation (Bryer et al. 2010).

For IHD patients, we used published registry data on acute coronary syndrome treatment in South Africa to determine practice patterns and thus cost ingredients (Schamroth 2012). We assumed 40% ST-elevation myocardial infarction and 60% non-ST-elevation acute coronary syndrome and distributed the proportion of patients receiving thrombolytics, cardiac catheterization, coronary stenting, and anticoagulation according to registry data. We assumed all patients underwent transthoracic echocardiography as well as chest radiography and electrocardiogram. We also included the cost of aspirin, clopidogrel, beta-blockers, and simvastatin according to the frequency of usage reported in the registry. Finally, we assumed that 8% of IHD patients would be eligible for coronary artery bypass graft surgery and thus added this to the total weighted average IHD cost.

For HHF patients, we followed a similar approach to the IHD methodology as registry data for heart failure in sub-Saharan Africa have recently been published (Damasceno et al. 2012). We assumed all patients with new-onset heart failure underwent echocardiography, chest radiography, electrocardiogram, and noninvasive stress testing to exclude ischemia. For inpatients in cardiogenic shock, we assumed dobutamine and nitrate infusion while in ICU. For all patients, we assumed diuretic infusion gradually tapered to oral therapy at discharge, as well as digoxin for some according to registry data. For outpatient therapy, we included the cost of angiotensin-converting enzyme inhibitors, beta-blockers, diuretics, digoxin, and spironolactone according to the frequency used in the heart failure registry. For both HHF and IHD, we did not consider include the potential cost of cardiac transplantation, since this is infrequently available.

For ESRD patients, we included the cost of renal ultrasound and urinalysis in the initial workup, as well as placement of a permanent dialysis catheter. We assumed all eligible patients would undergo hemodialysis three times weekly. We assumed all patients would be taking erythropoiesis-stimulating agents and phosphate binders. We did not consider the potential cost of renal transplantation, since this is infrequently available.

Finally, for the routine management of hypertension, we included ingredients listed in the latest South African hypertension guidelines (Seedat et al. 2012) including electrocardiogram, urine dipstick, serum chemistries and glucose. We assumed that 50% of patients would be on hydrochlorothiazide alone and the remainder on two drugs: hydrochlorothiazide and either of amlodipine or enalapril. Aside from antihypertensive use for specific CVD indications (e.g., enalapril for left ventricular dysfunction), we considered all hypertension costs separately from CVD treatment costs.

We assigned private uninsured patients the total cost of private care. We multiplied this total cost by 13.9% to obtain the average co-pay amount that insured patients would pay out-of-pocket based on the South African National Health Accounts (available at <http://www.who.int/nha/country/zaf/en/>).

**Table 1.** OOP costs of hypertension and CVD outcomes per payer category.

| **Payer category** | **Hypertension** | **Stroke** | | **IHD** | | **HHF** | | **ESRD** | |
| --- | --- | --- | --- | --- | --- | --- | --- | --- | --- |
| H0 | $0 | $0 | | $0 | | $0 | | $0 | |
| H1 | $5 | $24 | | $27 | | $22 | | $324 | |
| H2 | $29 | $360 | | $519 | | $252 | | $1661 | |
| H3 | $60 | $2043 | | $2240 | | $1523 | | $3402 | |
| Private insured | $12 | $532 | | $625 | | $392 | | $1701 | |
| Private uninsured | $87 | $3830 | | $4494 | | $2822 | | $23,643 | |
|  |  | |  | |  | |  | |  |

All costs are given in 2012 US dollars. Please see Supplement S2 for descriptions of the payer categories.

**Supplement S5. Univariate sensitivity analysis of key model inputs not provided in the main text.**

For salt intake, blood pressure reduction regression coefficients, CVD death rates, and CVD hazard ratios, we used confidence intervals provided in the original studies. For the stroke and HHF CFRs, we also used ranges provided in the original studies. No CFR ranges were available from the studies on IHD or ESRD, so we used the published difference between high-income European CFRs and African CFRs to establish the high and low boundaries for IHD, and used proportional variation in HHF CFRs from the literature to establish the high and low boundaries for ESRD. For costs, we used a lower bound of 50 percent of the original cost and an upper bound of 200 percent of the original cost.


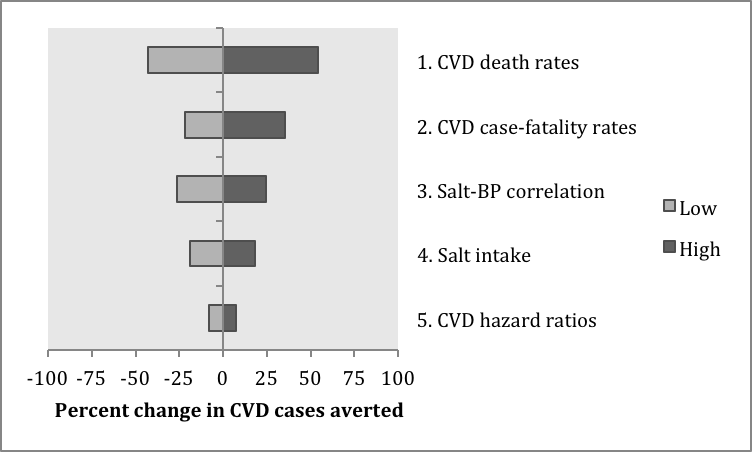


**Figure 1.** Univariate sensitivity analysis: change in cardiovascular disease (CVD) cases averted due to changes in key model inputs. The tornado diagram demonstrates the percent increase (dark grey) or decrease (light grey) in CVD cases averted when various model inputs are increased or decreased, respectively. BP = blood pressure.


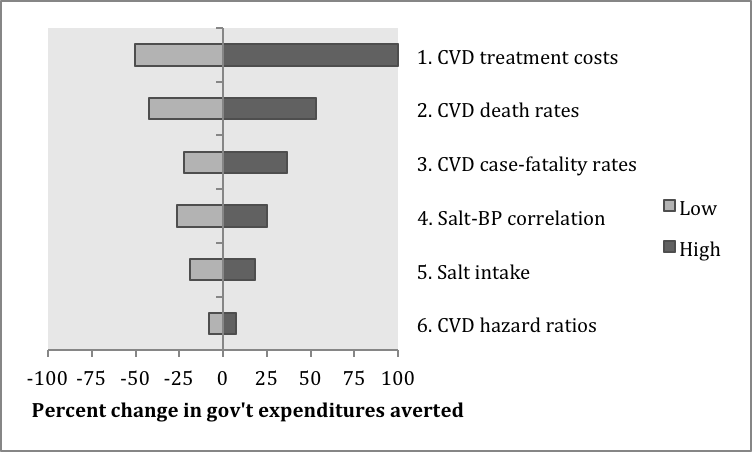


**Figure 2.** Univariate sensitivity analysis: change in total government subsidies averted due to changes in key model inputs. The tornado diagram demonstrates the percent increase (dark grey) or decrease (light grey) in subsidies averted when various model inputs are increased or decreased, respectively. CVD = cardiovascular disease, BP = blood pressure.


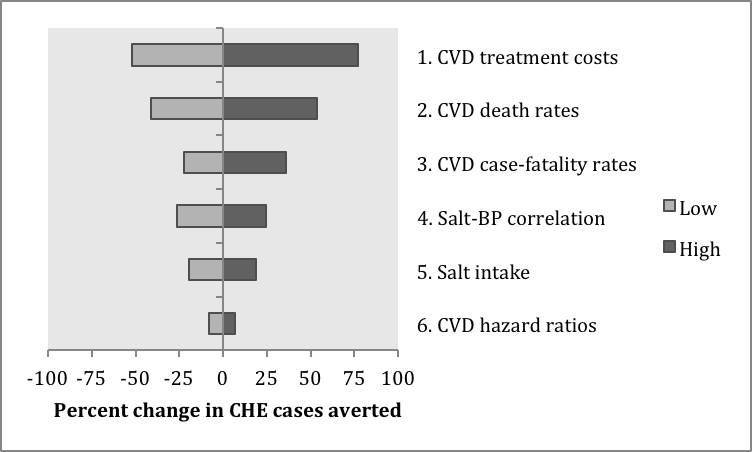


**Figure 3.** Univariate sensitivity analysis: change in cases of catastrophic health expenditure (CHE) averted due to changes in key model inputs. The tornado diagram demonstrates the percent increase (dark grey) or decrease (light grey) in CHE cases averted when various model inputs are increased or decreased, respectively. CVD = cardiovascular disease, BP = blood pressure.

**References for the Supplementary Materials**
